# Trend and Sociodemographic Correlates of Cesarean Section Utilization in Nepal: Evidence from Demographic and Health Surveys 2006-2016

**DOI:** 10.1155/2021/8888267

**Published:** 2021-05-03

**Authors:** Kiran Acharya, Yuba Raj Paudel

**Affiliations:** ^1^New ERA, Rudramati Marg, Kathmandu, Nepal; ^2^School of Public Health, University of Alberta, Edmonton, Canada

## Abstract

**Background:**

Addressing inequalities in accessing emergency obstetric care is crucial for reducing the maternal mortality ratio. This study was undertaken to examine the time trends and sociodemographic correlates of cesarean section (CS) utilization in Nepal between 2006 and 2016*. Methods.* Data from the Nepal Demographic and Health Surveys (NDHS) 2006, 2011, and 2016 were sourced for this study. Women who had a live birth in the last five years of the survey were the unit of analysis for this study. Absolute and relative inequalities in CS utilization were expressed in terms of rate difference and rate ratios, respectively. We used multivariable regression models to assess the CS rate by background sociodemographic characteristics of women.

**Results:**

Age and parity-adjusted CS rates were found to have increased almost threefold (from 3.2%, 95% CI: 2.1-4.3 in 2006 to 10.5%; 95% CI: 8.9-11.9 in 2016) over the decade. In 2016, women from mountain region (3.0%), those from the lowest wealth quintile (2.4%), and those living in Karnali province (2.4%) had CS rate below 5%. Whereas women from the highest wealth quintile (25.1%), with higher education (21.2%), and those delivering in private facilities (37.1%) had CS rate above 15%. Women from the highest wealth quintile (OR-3.3; 95% CI: 1.6-7.0) compared to women from the lowest wealth quintile and those delivered in private/NGO-run facilities (OR-3.6; 95% CI: 2.7-4.9) compared to women delivering in public facilities were more than three times more likely to deliver by CS.

**Conclusion:**

To improve maternal and newborn health, strategies need to be revised to address the underuse of CS among poor, those living in mountain region and Province 2, Lumbini province, Karnali province, and Sudhurpaschim province. Simultaneously, there is a pressing need for policies, guidelines, and continuous monitoring of CS rates to reduce overuse among rich women, women with higher education, and those giving childbirth in private facilities.

## 1. Background

Caesarean section (CS) is one of the vital interventions to save lives of mothers and babies at the time of life-threatening complications during pregnancy and child birth [1]. There is a growing public health concern that population-based CS rates in some countries have reached above the WHO recommended level [2, 3]. Nonmedical factors such as monetary incentives for health care providers and patient preferences are suggested to be key factors for unnecessary CS [4].

Government of Nepal (GoN) aims to reduce maternal and child mortality at the critical time of childbirth by ensuring basic emergency obstetric and newborn care and comprehensive emergency obstetric and newborn care services to all women who need it [5]. Nepal Health Sector strategy has highlighted equitable access to health services and leaving no one behind as one of the major foci of health sector [6]. Additionally, in line with sustainable development goals, GoN strives to reduce maternal mortality ratio to less than 70 per 100,000 live births and to reduce newborns and children deaths due to preventable causes to below one percent [7]. It is critical to have an easy access to emergency obstetric and newborn care to achieve these goals.

Nepal's Safe Motherhood Program has been operational since 1997 with the aim of reducing maternal and newborn mortality. Nepal introduced maternity incentive scheme in 2005 (popularly known as *Aama Program*) to reduce financial barriers to reach health facility for institutional delivery. Additionally, GoN has rolled out free delivery care including CS nationwide by abolishing all user fees since 2009 from accredited facilities [8]. Furthermore, current Aama Program provides a cash incentive of NPR 3,000 (USD 30 approximately) in mountain districts, NPR 2,000 (USD 20 approximately) in Hill districts, and NPR 1,000 (USD 10 approximately) in Terai districts to women delivering at health institutions [9]. This also includes NPR 800 (USD 8 approximately) for women who completed four antenatal cares as per GoN protocol. Health facilities also receive a case-based payment for providing free delivery according to the type of delivery (normal deliveries: USD 10 for HFs below 25 beds and USD 15 for HFs above 25 beds; complicated deliveries: USD 30 and CS: USD 70) [10–12]. Moreover, GoN has established an emergency referral fund in order to airlift women from geographically remote regions to referral centers in case of complications [5]. As a result of these demand-side and supply-side policies, institutional deliveries in Nepal have considerably increased from 9% in 1996 [13] to 57% in 2016 [14]. However, given the geographical diversity and sociocultural barriers, reaching poor, marginalized, uneducated, and women living in the remote and rural areas is still a big concern [15, 16]. Furthermore, Mehata et al. showed significant inequalities in CS in Nepal by place of residence, wealth quintile, age of the mother, educational status, and caste/ethnicity [17].

CS are not easily accessible to women from marginalized sections of the communities and those living in remote areas even when there is a strong medical indication [18]. Simultaneously, unnecessary use is common in private hospitals [3], urban areas [19], and among higher educated mothers [20]. Although an increasing number of private and community hospitals are now implementing Aama program, charging fees for CS is common [21]. In resource-poor settings such as Nepal, poor quality of obstetric care (lack of support and monitoring of child birth process, lack of pain management) resulting in less confidence to health care is thought to have increased demand for CS among upper and middle class women [22, 23]. Since private hospitals in Nepal are hitherto largely unregulated, high CS rate in private hospitals may be the result of “on-demand” or “provider-initiated” for monetary incentives [16, 24]. Additionally, there is a poor readiness of care in public hospitals to provide CS as indicated by considerable time lag between indication for CS and performance of CS [25] and maternity service users' least satisfaction with the cleanliness of health facilities [26]. Another longitudinal study from Western Nepal reported lower scores on amenities and interpersonal aspects in public hospitals compared to private hospitals from service user perception [27].

Although both underuse and overuse were associated with poor health outcomes for mothers and newborn, determining optimal CS rate at the population level is a herculean task [28]. WHO's new statement released in 2015 recommended that the CS rates should be below 10% [28]. Besides poor health outcomes, CS is associated with high cost to the health care system and to the families [29]. Furthermore, as in other settings [30], surge in CS rate has received increasing attention in Nepal on the grounds of women's rights, overmedicalization of birth, and abuse of technology [24]. Therefore, it is imperative to have an optimal CS rate at a population level while protecting the rights of women to have CS when there is a medical indication. Hence, it is important to monitor service utilization pattern and inequalities by population groups to assess program and policy outcomes. Therefore, by utilizing the most recent household level data from last three rounds of DHS surveys we present the trend and social determinants of utilization of CS in Nepal.

## 2. Methods

### 2.1. Data Source

We used data from three rounds of the Nepal Demographic and Health Survey (NDHS) conducted in 2006, 2011, and 2016. New ERA, a local research firm in Nepal, was responsible in implementing all these surveys under the leadership of Ministry of Health and population (MoHP) and technical assistance from ICF. Briefly, NDHS collected nationally representative data every 5 years on a broad range of issues including fertility, reproductive and maternal health, nutrition, and child health. In each round, a nationally representative sample of households was obtained using stratified cluster sampling approach. NDHS 2006 and 2011 used two-stage stratified cluster sampling to select households. Stratification was done by place of residence (urban/rural). In the first stage, Primary Sampling Units (PSUs) were selected using probability proportional to size. In the second stage, households were selected using systematic sampling from individual PSU in rural areas. However, three-stage stratified cluster sampling technique was used for selecting households in urban areas for NDHS 2016; i.e., in the first stage, PSUs were chosen by probability proportional to size followed by random selection of enumeration areas (EA) from PSUs; in the second stage and in the third stage, households were selected systematically from selected EAs. Details of the survey design and data collection procedure are available in respective survey reports [14, 31, 32]. Women aged 15-49 years who had a live birth within 5 years preceding the survey comprised the unit of analysis for the current study. In the case when there was more than single birth within the five-year period, we considered the most recent birth in our study.

### 2.2. Sample Size and Variables

The sample size for the analysis is most recent birth of eligible women aged 15-49 years who had a live birth five years preceding the surveys. This includes 5545 live births in 2006, 5391 live births in 2011, and 5060 live births in 2016. The dependent variable for this study was whether the last birth of a woman aged 15-49 years occurred by CS, represented with the same questions in all surveys, such as “Was the (name of the last child) delivered by caesarian section? The responses were recorded as a binary variable either 1 meaning “Yes” or 0 meaning “No.”

Based on the available literature [33–35], the independent variables selected for this study include women's age categories; parity (2 or less, 3-4, and more than 4); women's education: no education, primary (classes 1-5), some secondary (classes 6-9), secondary, and higher (class 10 and above); women's current working status (currently working, not working, and aside from her own housework, have done any work in the last seven days preceding the survey); wealth index; ecological region: Mountain (northern part of Nepal), Hill (midhilly region), and Terai (low land region) (i.e., Terai region is more accessible in terms of road and services followed by Hills and Mountains); provinces (Province 1, Province 2, Bagmati Province, Gandaki Province, Lumbini Province, Karnali Province, and Sudhurpaschim Province); place of residence (urban/rural); ethnicity; and place of delivery. The wealth index used for this study was calculated by principal component analysis based on the easy-to-collect data on a household's ownership of selected assets, such as televisions and bicycles; materials used for housing construction; and access to water and sanitation facilities. Households were then categorized to the lowest, second, middle, fourth, or the highest quintile group [33, 36]. Since there was no classification of the provinces for earlier NDHS and also there was no variable classified in NDHS 2006 and 2011, we have classified them using the verified comparability of clusters across each survey. This was done by using open access Global Positioning System (GPS) code of the clusters [37].

### 2.3. Data Analysis

Initially, we computed crude CS rates by women's sociodemographic characteristics. Then, we estimated the adjusted CS rates standardized for parity and age through a direct standardization method using the samples of NDHS 2016, as the standard population. We also estimated confidence intervals of parity- and age-adjusted CS rates. The CS rates were adjusted by parity and age using “stdweight,” and the standardization was done using command “stdize” in STATA. Similarly, to measure the inequalities, two inequality indicators—the ratio between the highest rank and the lowest rank [38, 39] and difference between the highest rank and the lowest rank (rate difference) were calculated for this study. The Rate ratio indicator links the level of health or use of health services between the highest and lowest ranks. Rate difference provides an absolute difference in prevalence of caesarian section between the highest and lowest ranks. To some extent, these two indicators, i.e., absolute inequality in terms of rate difference and relative inequality in terms of rate ratio, were done for each of the survey periods [39–42]. Presenting both relative and absolute measures of inequalities is important for increasing transparency and to provide unbiased evidence for policy making [43]; however, these two frequently used measures of inequality are easy to understand, but comparisons are limited to two extreme groups rather than covering the full population variety [38, 39]. We conducted multivariable regression analysis adjusting for women's age and parity to estimate the odd ratios of CS according to the women's characteristics. Variance inflation factor (VIF) was used to measure the correlation between the independent variables before running the regression model. Since no collinearity was detected, all variables were included in the regression model. Further, model fit was assessed using STATA's postestimation goodness of fit test. The level of statistical significance was set to 0.05. Although recent guideline has suggested CS rate of 10% as adequate at population level [28], we used 5% as a minimum rate and 15% as a maximum rate to interpret underuse and overuse of CS based on previously accepted standards [44, 45]. We used sampling weights to adjust for variations in the selection probabilities and interviews among respondents, and “svyset” command was used to account for complex survey design and to provide unbiased estimate. All analyses were performed using STATA version 15.0.

### 2.4. Ethical Considerations

Nepal DHS surveys were reviewed and approved by Nepal Health Research Council (NHRC) and the Institutional Review Board of ICF. The interviewers pursued informed consent from the women before the interviews as per the guideline of NHRC. We used deidentified publicly available data upon the request from DHS program (https://dhsprogram.com/Data/terms-of-use.cfm). This is the secondary analysis, and the publicly available datasets did not include individual identifiers and thus did not require ethics approval.

## 3. Results

The analysis revealed most of the women were 20-29 years old and constituted about two-thirds of the total population in all three surveys. The proportion of women with higher education increased remarkably from 6.2% in 2006 to 21.4% in 2016. Inverse to higher education, the proportion of working women decreased from 69.0% in the 2006 to 49.4% in the 2016. Concurrently, we found little difference in the distribution of women's wealth index. Among the respondents, less than 20% belonged to the fourt hand highest quintile group in all three surveys. During 2006 to 2011, more than 80% of the women lived in rural areas while it decreased to 46.0% in 2016. About one-third of the women belonged to Newar/Janjaties in all surveys. The proportion of women giving birth at health facilities (public and private sectors) increased rapidly over the decade. The proportion women giving birth at public facility increased by more than threefolds, from 13.1% in 2006 to 43.1% in 2016, while the proportion of birth in private facilities also doubled during the same period from 4.6% to 10.8% (Table 1).

Crude CS increased from 2.7% in 2006 to 9.0% in 2016, with large and increasing absolute disparities in CS according to women's sociodemographic characteristics (Table 2). A higher education level was associated with a markedly higher CS rate in all three surveys, specifically; in 2016, this rate was at 19.5%. Furthermore, higher CS rates (28.2% in 2016) were observed among the women from the highest wealth quintile and the lowest rate (2.4%) among women from the lowest wealth quintile. The disaggregation of the data by province showed that Bagmati province had persistently higher CS rate (6.0%, 8.3%, and 17.4% in 2006, 2011, and 2016, respectively) followed by Gandaki province (1.7%, 4.0%, and 16.7% in 2006, 2011, and 2016, respectively). Similarly, women living in urban areas had higher CS rates than women did in rural areas, although the absolute difference reduced during 2016 compared with 2006 and 2011. A greater difference was seen in the utilization of CS between public and private health facilities. This was true for all three rounds of the NDHS. The CS rates in the public and private sectors were 13.9% and 18.4%, respectively, in 2006; 8.6% and 25.4%, respectively, in 2011; and 12.1% and 34.8%, respectively, in 2016. Among the different caste/ethnic groups, the Brahmin/Chhetri had a higher CS rate with an increase from 4.4% in 2006 to 11.3% in 2016 followed by Newar/Janajati from 2.3% in 2006 to 10.2% in 2016. There was an increasing trend of CS among working and nonworking women.

It is interesting to note that the proportion of CS among those delivered in HFs increased slightly from 15.1% in 2006 to 16.7% in 2016 (Table 2).

Age- and parity-adjusted CS rates increased from 3.2% in 2006 to 10.5% in 2016 representing almost a threefold increase during the 10-year period (Table 3). The greatest increase in CS was seen among women with a higher educational level, those in the highest wealth quintile, and women who delivered in private facility. Similar patterns of socioeconomic differences were observed among women living in Province 1, Bagmati province, and Gandaki province. The absolute inequality in CS rate by maternal educational status increased from 9.7% in 2006 to 15.2% in 2016 (change of 5.5 percentage points). Similarly, the absolute inequality in CS rate by wealth quintiles more than doubled from 10.3% in 2006 to 22.7% in 2016. Furthermore, absolute inequality also increased for ecological region and provinces of Nepal. Notably, the absolute difference in CS rate between public and private facilities increased from 3.5% to 24.5%.

Relative inequality among women decreased in most of the sociodemographic characteristics and geographical distribution. However, the relative inequality increased in the provinces (from 5.0 in 2006 to 7.3 in 2016) and by place of delivery (1.3 in 2006 to 2.9 in 2016). In 2016, the highest absolute inequalities were observed for the place of delivery followed by wealth status, maternal education, and province. The highest relative inequality was observed for wealth quintile, province, ecological region, and maternal education. Of the total deliveries at public facilities, those conducted by CS remained almost constant (12.3% in 2006 and 12.6% in 2016). Meanwhile, among the total deliveries conducted in private facilities, there was a sharp rise in CS from 15.8 % in 2006 to 37.1% in 2016 (Table 3).

Table 4 shows age- and parity-adjusted odds ratios of CS using multivariable regression analysis. The likelihood of CS was three times higher among women belonging to the highest quintile group in 2016 (OR 3.3, 95% CI: 1.6-7.0) compared to the lowest wealth quintile. Similarly, women who delivered in private facilities were more than three times likely to have CS in both 2011 (OR: 3.2, 95% CI: 2.2-4.6) and 2016 NDHS (OR: 3.6, 95% CI: 2.7-4.9) surveys compared to those delivered in public facilities.

## 4. Discussion

Vaginal birth is associated with positive health outcomes for both mothers and infants and long-term physical and mental well-being of children. It is associated with improved breastfeeding, greater mother and infant bonding and is less costly for the family and the health care system in comparison to CS delivery [46–48]. Furthermore, vaginal birth is associated with early attainment of development milestones and lower risk of obesity among children [49]. Women giving vaginal birth consider it a symbol of achievement, empowerment, and elation [50]. Despite wide ranging benefits of vaginal birth, 10-15% CS rate is considered acceptable at population level to save lives of mothers and infant from birth-related complications [45]. In this study, we examined the trend of CS rate in Nepal and inequalities in its utilization by geography (Mountain, Hill and Terai); place of residence (urban/rural, Province); population characteristics (educational status, wealth quintile, caste/ethnicity, occupational status); and place of delivery. We found that overall CS rate in Nepal in 2016 (9%) was below the WHO's maximum limit of 15% and was much lower than in China [51, 52], Bangladesh [29], and Brazil [30]. The current analysis revealed more than threefold increase (from 3.2% and 95% CI: 2.1-4.3 in 2006 to 10.5% and 95% CI: 8.9-11.9 in 2016) in age and parity-adjusted CS rate over the decade. The rising trend in CS found in our study is consistent with the experience of other low and middle income countries [34, 53]. This increase is largely due to increase in the proportion of institutional deliveries. Between 2006 and 2016, the proportion of institutional deliveries (at public and private/NGO run facilities) in Nepal increased from 17.7% to 54% [14, 31]. Similarly, expansion of 24 hour birthing centers and emergency obstetric care in selected facilities has eased identification of possible complications and referral to higher facilities [5]. Furthermore, in the fiscal year 2016/17, CS service was expanded to 72 districts [54], which was available only in 45 districts until 2009/10 [55] out of then 75 districts in Nepal. Additionally, GoN has implemented demand side financing scheme all over Nepal since 2009 to remove financial barriers to receive institutional deliveries, including CS when needed [5]. While service expansion and demand side financing played a major role in increasing CS rate in Nepal, the role of patients' preferences (choice of private facility over public facility, lower pain, and choice of exact moment for delivery based on astrological belief) and provider preferences (save time, manage scheduling, increase earning, and avoid litigation/harassment) [53] cannot be overlooked. Moreover, increase in maternal age at first birth, growing proportion of pregnant women with the history of CS, and improvement in CS procedure may be associated with increase in CS rates as seen in other settings [56, 57].

Current analysis also revealed that while CS rate among the highest quintile increased (from 10.8% in 2006 to 25.1% in 2016), it declined among women from the lowest wealth quintile after 2011 initially increasing from the level of 2006. Additionally, both the absolute and relative inequalities were larger by wealth quintile (rate difference: 22.7, rate ratio: 10.5) compared to maternal educational status in 2016 (rate difference: 15.2, rate ratio: 3.5). These findings clearly show that significant barriers exist for women from the lowest wealth quintiles to access CS despite the existence of demand side financing scheme that cover transportation incentives and free delivery. A study by Acharya et al. showed that average hidden cost (food and drinking, transportation, cost for childcare, and cost for cloths) for delivering by CS in tertiary hospitals in Western Nepal was USD 321.6 [48], and the cost was significantly higher to deliver at a private hospital.

Furthermore, women with higher educational level (21.2%; 95% CI: 14.7-27.8) had a higher CS rate than women with no education (6.3%; 95% CI: 3.5-9.0) after adjusting for maternal age and parity (Table 3). Fear of labor pain, choice of a date or time thought to be auspicious, and concern with their sexual life after vaginal delivery [58] were identified as some of the factors associated with choice of CS delivery among educated women. Other possible reasons for high proportion of CS delivery among higher educated women may be associated with their higher income level and affordability [59]. Additionally, educated couples may be ignored by health educators, and hence, they might have less information about negative consequences of CS delivery [59]. Moreover, educated couples are more likely to be employed, and elective CS may be perceived to be safer and less interfering with work schedule [60]. However, the association with education disappeared in multivariable regression analysis (Table 4). We previously found inequalities in full vaccination of children were larger by household wealth quintiles than by maternal educational status [37]. Altogether, these findings suggest economic barriers for service utilization to be stronger than maternal educational-related barriers in Nepal for maternal health and immunization service utilization. Between 2006 and 2016, rate difference by maternal education increased while rate ratio decreased from 8.5 in 2006 to 3.5 in 2016. Literature suggests that rate ratio decreases as the service coverage increases from an overall lower level, whereas rate difference increases as the coverage reaches from low level to intermediate level [41].

Multivariable regression analysis also detected significant inequalities by provinces on the use of CS in comparison to Province 1. In addition to demand side factors (sociocultural factors, wealth status, and educational factors), unavailability/irregular functionality of birthing centers, emergency obstetric services, and geographical difficulties might have contributed to lower service utilization in some provinces and remote areas.

We found that women delivering in private facility (37.1, 95% CI: 30.5-43.7) have much higher CS rates than the WHO's recommended level. Age and parity-adjusted CS rate in private facilities more than doubled from 15.8% in 2006 to 37.1% in 2016 while it remained almost constant (12.3% in 2006 to 12.6% in 2016) in public facilities over ten years. In 2016, larger increase in the proportion of deliveries in public facilities than in private facilities between 2006 and 2016 and constant CS rate in public facilities led to a slight overall increase in CS rate among deliveries conducted at HFs (public+private). In fact, women delivered in private/NGO-run facilities were nearly 4 times more likely to have CS (adjusted OR 3.6; 95% CI: 2.7-4.9) compared to women delivering in public facilities. Such a high proportion of CS in these facilities suggest that nonmedical factors (economic gain, manage scheduling, or others) might have motivated health care providers to perform CS, especially in private HFs. The rise in CS in Brazil especially among women of upper and middle class delivering in private sector was found to be induced by provider rather than by consumer preferences [4]. The authors concluded that obstetricians perceived CS to be safer than vaginal delivery, or they might assume women in private facilities would prefer CS without exploring women's choices and expectations. Alternatively, it was suggested that given the staff constrains and workload issues, CS might be an easier option to manage schedules for managers and service providers [4]. Having said that, maternal/family preference for CS in order to avoid pain or due to the perception that CS are safer than vaginal delivery may also have contributed to high CS rates in private facilities as reported in other studies [61–63]. Relatively constant CS rate in public facilities indicates rational use of CS in these facilities. Since there is no or less financial incentive to service providers when delivering by CS in public facilities, they may have conducted CS as an emergency life-saving procedure. Previous studies from Nepal showed that public hospitals offering maternity care have poor cleanliness [26] and have less amenities and poor interpersonal communication from service providers in comparison to private hospitals [27]. Additionally, maternity wards in public hospitals are overcrowded [64]. Despite perceived lower quality of care, public hospitals in Nepal attract more than three-quarters of all deliveries due to their reputation and perceived higher technical quality [25, 27] and lower cost of health care compared to private hospitals.

## 5. Implications

On the basis of current findings and earlier findings by Acharya et al. showing significant hidden cost to utilize CS [48], the maternity incentive program needs to be improvised in order to increase coverage and efficiency by targeting poor women from remote areas/mountain region/Karnali province where there is less access. Women from disadvantaged communities might benefit from support to cover hidden costs (transportation, food, and lodging) associated with CS delivery. Although, Ministry of Health has established emergency referral fund to airlift women from poor, Dalit, Janajati, geographically disadvantaged, and from mountainous region to transport to a higher facility in case of life-threatening maternity complications, the use of this fund is very low. Only nine women in FY 2017/2018 used this fund to be air lifted [5]. There is a need of an in-depth study to understand barriers to use emergency referral funds.

It is beyond the scope of this study to determine the cause for high CS in private facilities. However, such a high CS rate in private/NGO-run facilities in Nepal warrants an appropriately defined quality assurance mechanism [65]. Appropriate government policies and clinical practice protocols should be enacted to achieve an optimal CS rates. Increasing rates of CS have created a huge financial burden for Bangladesh's health care system [29]. Nepal's Ministry of Health should commission a study on an economic burden of CS in Nepal may be a starting point to understand its significance. Quality Assurance section under Management Division in collaboration with Family Welfare Division should implement a contextual and validated tool such as Robson classification to support health workers to assess need and monitor the use of CS [66]. Monitoring of the CS rate is necessary to ensure those who need the service are receiving and equity in health care use is achieved. Further, it can minimize provider-induced CS for the sake of money. Additionally, ensuring continuous support (emotional support, information support, and advocacy of her wishes) for women during childbirth [67], enhancing quality of obstetric care by ensuring adequate pain management, and use of technology can build up women's confidence in obstetric care and decrease maternal demand for CS [23]. Furthermore, the findings of this study clearly indicate a need to strengthen public hospitals and their capacity to provide CS delivery in mountain region, Province 2, Lumbini province, Karnali province, and Sudhurpaschim province. As given, low readiness of care to provide timely CS [25], less amenities and cleanliness, and poor communication skills of staff and public hospitals [26, 27] need service strengthening to ensure quality of care.

Some studies have indicated that midwife-led maternity care can reduce CS rates including the maternity costs [68]. In Nepal, maternity care in hospitals is obstetrician led and it is high time to consider promoting physiological birth through midwife led maternity care [24]. There is a need to scale up current midwifery education to improve maternity care as well as to reduce overburden of normal deliveries in tertiary care facilities.

Although the overall CS rates in Nepal are within the WHO standard [15], due attention is necessary to monitor the increasing CS rate (crude CS rate from 2.7% in 2006 to 9% in 2016) and appropriate measures should be in place to curb the increasing trend. On the other hand, women from the poorest wealth quintiles need special targeting. Furthermore, there is a need to expand CS in mountain districts and some provinces (Province 2, Lumbini province, Karnali province, and Sudhurpaschim province) and improve service quality in public hospitals to reduce inequality in service utilization.

### 5.1. Strengths and Limitations

We used high-quality nationally representative data to assess trend and inequalities in CS. However, DHS survey lacks clinical data to evaluate appropriateness of CS. However, it is unlikely that clinical factors drive these variations. Furthermore, supply side variables and variables related to sociocultural norms and beliefs were not available. Therefore, future studies can utilize supply side data from national surveys such as Nepal Health Facility Survey [69] for combining with DHS data to get a wider picture of the factors affecting CS utilization [70]. Furthermore, qualitative studies are needed to understand service users' and providers' perspectives determining CS utilization.

## 6. Conclusion

In this paper, we found age and parity-adjusted CS rate in Nepal to have increased almost threefold within the ten-year period. CS rate was consistently high among women from the wealth quintiles and those delivering in private sector. This study also reveals that absolute inequality expressed as rate difference was increasing by maternal educational status, household wealth quintile, and province, whereas relative inequality expressed by rate ratio has decreased over time for maternal educational status and wealth quintile. There was larger inequality in CS utilization by wealth quintile than maternal educational status. Therefore, strategies targeting mothers from the poor households, those from mountain region, and selected provinces (Province 2, Lumbini province, Karnali province, and Sudhurpaschim province) need to be in place along with improved service readiness in public hospitals. High CS rate in private facilities need to be closely monitored to ensure optimal use of CS delivery.

## Figures and Tables

**Table 1:** Sociodemographic characteristics and the use of cesarean section from the Nepal Demographic and Health Surveys (NDHS) in 2006, 2011, and 2016.

Characteristics	NDHS 2006		NDHS 2011		NDHS 2016	
	(*N* = 5545)		(*N* = 5391)		(*N* = 5060)	
	Frequency	%	Frequency	%	Frequency	%
*Maternal age (years)*						
15-19	368	6.6	381	7.1	391	7.7
20-24	1,931	34.8	1,802	33.4	1,665	32.9
25-29	1,742	31.4	1,736	32.2	1,780	35.2
30-34	785	14.2	845	15.7	806	15.9
35-39	465	8.4	402	7.5	297	5.9
40-44	196	3.5	169	3.1	97	1.9
45-49	58	1.0	57	1.1	24	0.5
*Parity*						
2 or less	3,132	56.5	3,293	61.1	3,472	68.6
3-4	1,459	26.3	1,405	26.1	1,161	22.9
More than 4	954	17.2	693	12.9	427	8.4
*Education*						
No education	3,343	60.3	2,550	47.3	1,733	34.3
Primary	1,009	18.2	1,079	20.0	1,019	20.1
Some secondary	848	15.3	1,039	19.3	1,226	24.2
Secondary and higher	345	6.2	723	13.4	1,082	21.4
*Current working status*						
Working	3,824	69.0	2,961	54.9	2,498	49.4
Not working	1,721	31.0	2,431	45.1	2,562	50.6
*Wealth index*						
Lowest	1,412	25.5	1,390	25.8	1,082	21.4
Second	1,180	21.3	1,182	21.9	1,072	21.2
Middle	1,132	20.4	1,133	21.0	1,121	22.2
Fourth	983	17.7	938	17.4	1,036	20.5
Highest	838	15.1	748	13.9	748	14.8
*Residence*						
Urban	677	12.2	503	9.3	2,730	54.0
Rural	4,868	87.8	4,888	90.7	2,330	46.0
*Ecological region*						
Mountain	483	8.7	428	7.9	361	7.1
Hill	2,261	40.8	2,130	39.5	1,911	37.8
Terai	2,802	50.5	2,833	52.6	2,789	55.1
*Province*						
Province 1	920	16.6	1,135	21.0	819	16.2
Province 2	1,171	21.1	1,146	21.3	1,367	27.0
Bagmati Province	920	16.6	705	13.1	813	16.1
Gandaki Province	571	10.3	589	10.9	388	7.7
Lumbini Province	822	14.8	810	15.0	899	17.8
Karnali Province	341	6.1	401	7.4	338	6.7
Sudhurpaschim Province	800	14.4	605	11.2	437	8.6
*Caste/ethnicity*						
Brahmin/Chhetri	1,639	29.6	1,618	30.0	1,396	27.6
Terai/Madhesi other	685	12.4	558	10.4	1,021	20.2
Dalits	884	15.9	959	17.8	695	13.7
Newar/Janajati	1,934	34.9	1,892	35.1	1,573	31.1
Muslim/other	402	7.3	364	6.7	375	7.4
*Place of delivery*						
Public facility	724	13.1	1,399	26.0	2,183	43.1
Private facility/NGOs	256	4.6	506	9.4	548	10.8
Outside Nepal	NA	NA	NA	NA	171	3.4
Home	4,492	81.0	3,402	63.1	2,096	41.4
Others	73	1.3	85	1.6	61	1.2
*Cesarean section*						
Yes	148	2.7	248	4.6	457	9.0
No	5,397	97.3	5,143	95.4	4,603	91

Cesarean section was determined for the most recent birth of women aged 15-49 years who had a live birth five years preceding the surveys. All percentages are weighted, so the absolute number of participants does not perfectly correspond to percentages. NA: not available.

**Table 2:** Crude cesarean section (CS) rates (%) by sociodemographic characteristics from the Nepal Demographic and Health Surveys (NDHS) in 2006, 2011, and 2016.

Characteristics	NDHS 2006			NDHS 2011			NDHS 2016		
	Total births	CS	%	Total births	CS	%	Total births	CS	%
Total (*N*)	5545	148	2.7	5391	248	4.6	5060	457	9
*Maternal age (years)*									
15-19	368	16	4.2	381	14	3.5	391	25	6.3
20-24	1,931	47	2.4	1,802	64	3.6	1,665	142	8.5
25-29	1,742	56	3.2	1,736	93	5.4	1,780	163	9.2
30-34	785	17	2.2	845	52	6.1	806	88	11
35-39	465	3	0.6	402	18	4.4	297	35	11.8
40-44	196	9	4.4	169	7	4.4	97	4	3.6
45-49	58	0	0	57	<1	0.6	24	0	0
*Parity*									
2 or less	3,132	129	4.1	3,293	203	6.2	3,472	407	11.7
3-4	1,459	7	0.5	1,405	43	3.0	1,161	42	3.6
More than 4	954	11	1.1	693	2	0.3	427	8	1.8
*Education*									
No education	3,343	37	1.1	2,550	45	1.8	1,733	78	4.5
Primary	1,009	28	2.8	1,079	44	4.1	1,019	57	5.6
Some secondary	848	35	4.1	1,039	66	6.3	1,226	110	9
Secondary and higher	345	48	13.9	723	93	12.9	1,082	211	19.5
*Current working status*									
Working	3,824	52	1.4	2,961	82	2.8	2,498	193	8
Not working	1,721	96	5.6	2,431	166	6.8	2,562	264	10
*Wealth index*									
Lowest	1,412	11	0.8	1,390	14	1.0	1,082	26	2.4
Second	1,180	6	0.5	1,182	10	0.8	1,072	46	4.2
Middle	1,132	11	1.0	1,133	52	4.6	1,121	77	6.8
Fourth	983	19	2.0	938	67	7.1	1,036	97	9.4
Highest	838	100	11.9	748	106	14.1	748	211	28.2
*Residence*									
Urban	677	57	8.4	503	77	15.3	2,730	320	11.7
Rural	4,868	90	1.9	4,888	171	3.5	2,330	137	5.9
*Ecological region*									
Mountain	483	4	0.7	428	6	1.4	361	9	2.6
Hill	2,261	70	3.1	2,130	78	3.7	1,911	214	11.2
Terai	2,802	74	2.6	2,833	164	5.8	2,789	234	8.4
*Province*									
Province 1	920	19	2.1	1,135	74	6.5	819	104	12.7
Province 2	1,171	25	2.1	1,146	47	4.1	1,367	69	5.0
Bagmati Province	920	55	6.0	705	59	8.3	813	141	17.4
Gandaki Province	571	10	1.7	589	24	4	388	65	16.7
Lumbini Province	822	27	3.3	810	30	3.7	899	57	6.4
Karnali Province	341	5	1.4	401	4	1	338	7	2.2
Sudhurpaschim Province	800	7	0.8	605	11	1.8	437	13	3.1
*Caste/ethnicity*									
Brahmin/Chhetri	1,639	72	4.4	1,618	118	7.3	1,396	158	11.3
Terai/Madhesi other	685	15	2.2	558	33	6	1,021	73	7.1
Dalits	884	11	1.2	959	20	2.1	695	38	5.4
Newar/Janajati	1,934	45	2.3	1,892	64	3.4	1,573	160	10.2
Muslim/other	402	4	1.0	364	12	3.2	375	28	7.4
*Place of delivery*									
Public facility	724	101	13.9	1,399	120	8.6	2,183	265	12.1
Private facility/NGOs	256	47	18.4	506	129	25.4	548	191	34.8
Outside Nepal	NA	NA	NA	NA	NA	NA	171	1	0.8
Home	4,492	0	0	3,402	0	0	2,096	0	0
Others	73	0	0	85	0	0	61	0	0

Cesarean section was determined for the most recent birth of women aged 15-49 years who had a live birth five years preceding the surveys. All percentages are weighted, so the absolute number of participants does not perfectly correspond to percentages. The question on C-section was asked only of women who delivered in a health facility while analysis was based on the number of total births. NA: not available.

**Table 3:** Maternal age and parity-adjusted cesarean section (CS) rates (%) from the Nepal Demographic and Health Surveys (NDHS) in 2006, 2011, and 2016.

Characteristics	NDHS 2006	NDHS 2011	NDHS 2016
Overall adjusted CS rates (%)	3.2 (2.1-4.3)	6.6 (5.1-8.1)	10.5 (8.9-11.9)
*Education*			
No education	1.3 (0.5-2.1)	4.2 (2.7-5.7)	6.3 (3.5-9.0)
Primary	3.7 (1.1-6.3)	5.7 (2.7-8.7)	6.0 (3.5-8.6)
Some secondary	4.8 (2.4-7.2)	6.2 (3.9-8.5)	10.5 (7.7-13.3)
Secondary and higher	11.0 (7.8-14.2)	17.4 (11.3-23.5)	21.2 (14.7-27.8)
*Rate difference*	*9.7*	*13.2*	*15.2*
*Rate ratio*	*8.5*	*4.1*	*3.5*
*Current working status*			
Working	1.6 (0.8-2.3)	4.8 (3.4-6.2)	9.5 (7.6-11.4)
Not working	6.6 (4.2-9.0)	8.7 (6.6-10.7)	11.6 (9.4-13.9)
*Rate difference*	*5.0*	*3.9*	*2.1*
*Rate ratio*	*4.1*	*1.8*	*1.2*
*Wealth index*			
Lowest	1.4 (0.1-2.6)	2.7 (0.8-4.6)	2.4 (1.2-3.7)
Second	0.5 (0.03-0.9)	1.3 (0.1-2.6)	5.2 (3.0-7.4)
Middle	1.2 (0.09-2.4)	6.2 (4.0-8.4)	8.0 (4.9-11.1)
Fourth	1.4 (0.6-2.3)	8.7 (5.7-11.6)	9.3 (6.7-12.0)
Highest	10.8 (7.7-13.9)	13.4 (9.7-17.2)	25.1 (20.2-30.1)
*Rate difference*	*10.3*	*12.1*	*22.7*
*Rate ratio*	*21.6*	*10.3*	*10.5*
*Residence*			
Urban	8.0 (5.7-10.4)	14.8 (10.8-18.8)	12.4 (10.4-14.4)
Rural	2.0 (1.0-3.0)	5.3 (3.8-6.8)	7.0 (5.1-8.9)
*Rate difference*	*6.0*	*9.5*	*5.4*
*Rate ratio*	*4.0*	*2.8*	*1.8*
*Ecological region*			
Mountain	0.8 (0.02-1.6)	1.5 (0.6-2.4)	3.0 (1.1-4.9)
Hill	3.6 (2.0-5.2)	6.7 (4.9-8.5)	12.2 (9.9-14.5)
Terai	2.6 (1.6-3.6)	6.6 (5.1-8.1)	10.0 (7.9-12.1)
*Rate difference*	*2.8*	*5.2*	*9.2*
*Rate ratio*	*4.5*	*4.5*	*4.1*
*Province*			
Province 1	1.9 (0.6-3.1)	6.7 (4.0-9.4)	12.2 (8.8-15.6)
Province 2	2.1 (0.6-3.5)	6.6 (4.5-8.8)	6.8 (3.6-9.9)
Bagmati Province	6.0 (3.2-8.8)	12.1 (8.0-16.2)	16.4 (12.6-20.2)
Gandaki Province	2.2 (-0.05-4.5)	4.9 (2.3-7.6)	17.5 (12.5-22.4)
Lumbini Province	3.3 (1.6-5.1)	3.9 (2.1-5.7)	7.8 (5.0-10.5)
Karnali Province	3.0 (-0.1-6.1)	2.9 (2.0-3.7)	2.4 (1.3-3.5)
Sudhurpaschim Province	1.2 (-0.1-2.6)	2.8 (1.4-4.3)	5.7 (2.7-8.7)
*Rate difference*	*4.8*	*9.3*	*15.1*
*Rate ratio*	*5.0*	*4.3*	*7.3*
*Caste/ethnicity*			
Brahmin/Chhetri	5.2 (3.0-7.4)	8.3 (6.3-10.4)	11.8 (9.3-14.4)
Terai/Madhesi other	2.8 (0.8-4.9)	8.5 (4.0-13.0)	10.3 (6.1-14.6)
Dalits	0.9 (0.2-1.7)	2.7 (0.7-4.6)	7.8 (4.0-11.6)
Newar/Janajati	2.9 (1.0-4.9)	5.2 (3.3-7.1)	10.2 (7.8-12.6)
Muslim/other	0.8 (-0.2-1.8)	4.5 (0.5-8.6)	11.1 (4.1-18.2)
*Rate difference*	*4.4*	*5.8*	*4.0*
*Rate ratio*	*6.5*	*3.1*	*1.5*
*Place of delivery* ^∗^			
Public facility	12.3 (9.1-15.6)	11.1 (8.4-13.9)	12.6 (10.4-14.8)
Private facility/NGOs	15.8 (9.9-21.6)	26.4 (20.8-31.9)	37.1 (30.5-43.7)
Outside Nepal	NA	NA	0.7 (-0.6-2.0)
*Rate difference*	*3.5*	*15.3*	*24.5*
*Rate ratio*	*1.3*	*2.4*	*2.9*

^∗^Home deliveries were excluded from the analysis. Cesarean section was determined for the most recent birth of women aged 15-49 years who had a live birth five years preceding the surveys. The study sample of NDHS 2016 (*N* = 5060) was used as the standard population in the direct standardization. For each survey, categories with the highest and lowest CS rate were used to calculate the rate difference and rate ratios. NA: not available.

**Table 4:** Multivariable regression analysis of cesarean section (CS) from the Nepal Demographic and Health Surveys (NDHS) in 2006, 2011, and 2016.

Characteristics	NDHS 2006	NDHS 2011	NDHS 2016
	0R (95% CI)	0R (95% CI)	0R (95% CI)
*Education*			
No education	1.00 (reference)	1.00 (reference)	1.00 (reference)
Primary	1.0 (0.5-2.1)	1.6 (0.8-3.1)	0.7 (0.4-1.1)
Some secondary	0.5 (0.2-1.1)	1.5 (0.8-3.0)	0.6 (0.3-1.1)
Secondary and higher	0.6 (0.2-1.5)	1.4 (0.6-3.0)	0.7 (0.4-1.6)
*Current working status*			
Working	1.00 (reference)	1.00 (reference)	1.00 (reference)
Not working	2.1^∗∗^ (1.3-3.5)	1.5 (0.8-3.0)	1.1 (0.8-1.4)
*Wealth index*			
Lowest	1.00 (reference)	1.00 (reference)	1.00 (reference)
Second	0.2 (0.0-1.1)	0.3 (0.1-1.2)	1.0 (0.5-1.9)
Middle	0.3 (0.1-1.5)	0.9 (0.3-2.7)	1.1 (0.6-2.2)
Fourth	0.3 (0.1-1.8)	0.6 (0.2-1.8)	1.1 (0.6-2.0)
Highest	1.1 (0.2-6.3)	0.5 (0.2-1.6)	3.3^∗∗^ (1.6-7.0)
*Residence*			
Urban	1.00 (reference)	1.00 (reference)	1.00 (reference)
Rural	1.3 (0.7-2.3)	0.5^∗∗^ (0.3-0.8)	1.2 (0.9-1.6)
*Ecological region*			
Mountain	1.00 (reference)	1.00 (reference)	1.00 (reference)
Hill	0.9 (0.2-4.4)	1.0 (0.5-1.9)	1.5 (0.7-3.2)
Terai	1.0 (0.2-6.1)	1.5 (0.7-3.1)	1.6 (0.7-3.6)
*Province*			
Province 1	1.00 (reference)	1.00 (reference)	1.00 (reference)
Province 2	1.7 (0.6-4.7)	1.1 (0.5-2.2)	0.4^∗∗^ (0.2-0.8)
Bagmati Province	1.2 (0.5-3.2)	1.9^∗^ (1.1-3.4)	1.1 (0.7-1.9)
Gandaki Province	0.7 (0.1-3.9)	1.0 (0.5-1.8)	1.7^∗^ (1.1-2.8)
Lumbini Province	1.7 (0.7-4.2)	0.8 (0.4-1.6)	0.4^∗∗∗^ (0.3-0.7)
Karnali Province	0.7 (0.2-3.0)	0.7 (0.2-2.8)	0.5 (0.2-1.1)
Sudhurpaschim Province	0.7 (0.2-2.5)	0.5 (0.3-1.2)	0.4^∗∗^ (0.2-0.7)
*Caste/ethnicity*			
Brahmin/Chhetri	1.00 (reference)	1.00 (reference)	1.00 (reference)
Terai/Madhesi other	0.6 (0.2-1.7)	0.9 (0.4-2.0)	1.9^∗^ (1.0-3.6)
Dalits	1.1 (0.5-2.8)	0.7 (0.4-1.4)	1.3 (0.8-2.2)
Newar/Janajati	0.6 (0.3-1.2)	0.6^∗^ (0.4-0.9)	1.1 (0.8-1.5)
Muslim/other	0.3 (0.1-1.1)	0.7 (0.2-1.9)	1.1 (0.6-2.1)
*Place of delivery^#^*			
Public facility	1.00 (reference)	1.00 (reference)	1.00 (reference)
Private facility/NGOs	1.5 (0.9-2.8)	3.2^∗∗∗^ (2.2-4.6)	3.6^∗∗∗^ (2.7-4.9)
Outside Nepal	NA	NA	0.1^∗∗^ (0.0-2.5)

^#^Home deliveries were excluded from the analysis. Cesarean section was determined for the most recent birth of women aged 15-49 years who had a live birth five years preceding the surveys. Each variable in the model has been adjusted by all the given variables in this table including age and parity. NA: not available. ^∗∗∗^*p* < 0.001, ^∗∗^*p* < 0.01, and ^∗^*p* < 0.05.

## Data Availability

Data is easily accessible from the DHS program website (https://http://dhsprogram.com/Data/terms-of-use.cfm) upon the request.
